# A nomogram for predicting intra-operative conversion to endotracheal intubation during non-intubated spontaneous ventilation anesthesia in pulmonary resection: development of a risk prediction model in hypoxic and high-risk patients

**DOI:** 10.3389/fmed.2025.1709129

**Published:** 2025-11-28

**Authors:** Tao Lin, Bing Zhang, Lei Chen, Jialin Mei, Yongyue Zhu, Fei Gao, Jihao Dong, Yang Bao, Gaofeng Li

**Affiliations:** 1 Department of Cardiothoracic Surgery, Baoshan People’s Hospital, The Fifth Affiliated Hospital of Dali University, Baoshan, China; 2 Department of Thoracic Surgery, Yunnan Cancer Hospital (The Third Affiliated Hospital of Kunming Medical University and Yunnan Hospital of Peking University Cancer Hospital), Kunming, China

**Keywords:** non-intubated spontaneous ventilation anesthesia, pulmonary resection, intraoperative endotracheal intubation, prediction model, nomogram, risk assessment, non-intubated thoracic surgery

## Abstract

**Background:**

Non-intubated spontaneous ventilation anesthesia (NISVA) avoids complications associated with endotracheal intubation in pulmonary resection. However, intraoperative conversion to endotracheal intubation (IETI) occurs in significant numbers of patients. This study aimed to develop and validate a predictive model for IETI risk during NISVA -based pulmonary resection.

**Methods:**

This retrospective cohort study included 244 patients undergoing pulmonary resection under NISVA from January 2019 to December 2024. Patients were randomly divided into training (*n* = 170) and validation (*n* = 74) sets. Independent risk factors for IETI were identified using LASSO regression and multivariate logistic regression. A nomogram prediction model was constructed and validated using receiver operating characteristic (ROC) analysis, calibration curves, and decision curve analysis (DCA).

**Results:**

The IETI incidence was 45.49% (111/244). Five independent risk factors were identified: preoperative hypoxemia (OR = 2.973, 95% CI: 1.249–7.340), surgical site (lower lobe) (OR = 2.462, 95% CI: 1.055–5.827), Type of surgery (lobectomy) (OR = 3.600, 95% CI: 1.575–8.559), difficult airway (OR = 4.708, 95% CI: 1.984–11.87), and surgical duration ≥ 3 h (OR = 11.81, 95% CI: 4.617–33.96). The nomogram demonstrated excellent discrimination with AUCs of 0.889 (training) and 0.880 (validation). Calibration curves showed good agreement between predicted and observed probabilities. DCA indicated clinical utility across threshold probabilities of 5–85%.

**Conclusion:**

This novel nomogram accurately predicts IETI risk during NISVA -based pulmonary resection, enabling individualized preoperative assessment and optimization of anesthesia strategies. The model shows potential for improving surgical safety and patient outcomes in non-intubated thoracic surgery.

## 1 Introduction

Pulmonary resection is a key therapeutic modality for primary lung cancer and high-risk pulmonary lesions (1). Traditional surgical procedures typically require endotracheal intubation under general anesthesia, which may lead to a series of complications, such as airway injury (2) and ventilator-associated pneumonia (3). Non-intubated spontaneous ventilation anesthesia (NISVA) avoids the risks associated with endotracheal intubation. However, some patients may require conversion to endotracheal intubation (Intraoperative Endotracheal Intubation, IETI) due to intraoperative hypoxemia, hemodynamic instability, or when the surgical complexity exceeds what can be safely managed under spontaneous ventilation (4, 5). IETI can prolong the duration of surgery, increase the complexity and risk of anesthesia management, and may adversely affect the intraoperative stress response, postoperative pulmonary function recovery, and overall prognosis of patients (6–8). Therefore, accurately predicting patients who may require conversion to endotracheal intubation during pulmonary surgery under NISVA is of great clinical significance for optimizing preoperative assessment, formulating individualized anesthesia plans, and enhancing surgical safety.

Currently, research on the risk factors for conversion to endotracheal intubation during pulmonary surgery under NISVA remains relatively limited, and no effective predictive model has been established. This study aims to construct a predictive model for the risk of conversion to endotracheal intubation during pulmonary resection under NISVA, to identify the key factors influencing the need for conversion, and to provide a robust basis for clinicians, thereby further improving the efficacy and safety of NISVA in pulmonary surgery.

## 2 Materials and methods

### 2.1 Study design and setting

This single-center, retrospective observational cohort study was approved by the Institutional Review Board (IRB) of Baoshan People’s Hospital (IRB number: LL-2023-KYKT-14) and was conducted in strict accordance with the Declaration of Helsinki. Given the retrospective nature of the data analysis, written informed consent was waived. The study period was from January 2019 to December 2024, and included patients who underwent pulmonary resection under Non-intubated spontaneous ventilation anesthesia (NISVA).

### 2.2 Patient selection and grouping

#### 2.2.1 Inclusion criteria

Patients with primary lung cancer or high-risk pulmonary lesions confirmed by pathology who required surgical intervention and underwent elective thoracoscopic pulmonary resection under NISVA. Predicted difficult-airway risk score (DARS) 0–1 (i.e., no absolute difficult-airway features).

#### 2.2.2 Exclusion criteria

Patients with preoperative artificial airway establishment, missing key perioperative variables ≥ 5%, severe cardiovascular or cerebrovascular diseases, hepatic or renal failure, history of ipsilateral thoracic surgery, or those with special types of lung cancer or extensive pleural effusion and widespread lymph node metastasis.

**FIGURE 1:**
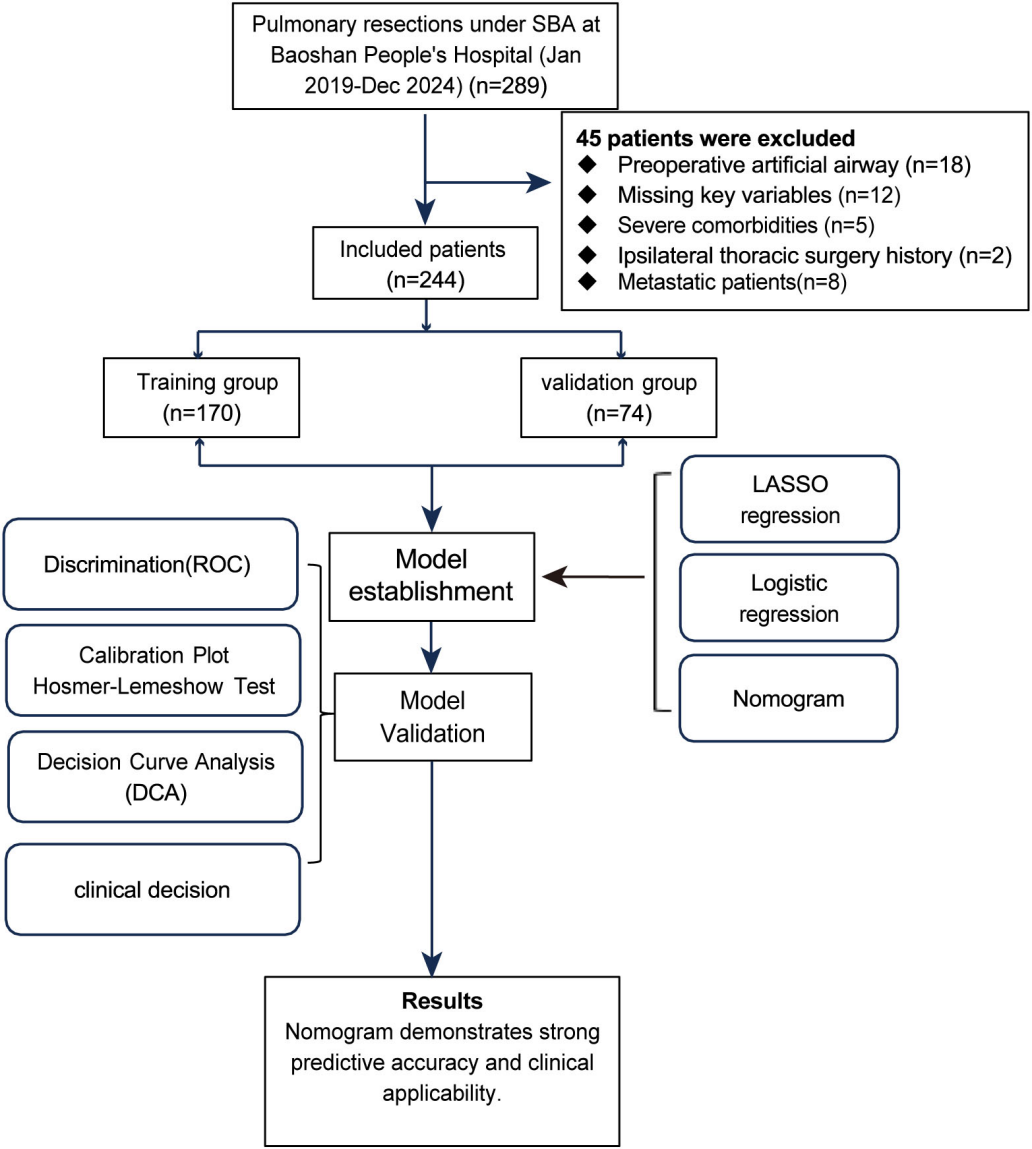
Research pathway diagram.

In this study, a simple random sampling method was used to divide patients into a training set and a validation set. First, 244 patients were numbered (1–244). A random number generator produced 170 distinct random numbers, and patients corresponding to these numbers were included in the training set. The remaining 74 patients formed the validation set. The ratio of the training set to the validation set was 7:3, a proportion commonly used in studies and suitable for the sample size of this study. The study flow is shown in Figure 1.

### 2.3 Data elements and definitions

The implementation of Intraoperative Endotracheal Intubation (IETI) adhered to the following three predefined criteria: (1) Refractory hypoxemia: SpO_2_ < 90% for > 5 min despite FiO_2_ = 1.0. (2) Hemodynamic collapse: MAP < 60 mmHg for ≥ 3 min after a 250 mL crystalloid bolus and optimization of anesthetic depth (continuous invasive radial-artery monitoring in all patients). (3) Surgical emergency: intra-operative circumstances mandating immediate intubation because extension to open thoracotomy or a larger incision could not be safely performed while spontaneous ventilation was maintained.

High-risk patients were defined as those with preoperative hypoxemia (SpO_2_ ≤ 92%), difficult airway (DARS ≥ 1), or requiring anatomical lobectomy. Hypoxemia was defined as arterial blood oxygen saturation (SpO_2_) ≤ 92% or arterial partial pressure of oxygen (PaO_2_) < 60 mmHg.

Anesthesia protocol: oral alprazolam 0.5 mg was administered 30 min before surgery. After entering the operating room, an ultrasound-guided bilateral paravertebral block was performed at the T4–T8 level; 0.375% ropivacaine 10 mL was injected on each side (total 20 mL). Sedation was maintained with target-controlled infusions of propofol (effect-site concentration 1.0–1.5 μg mL^–1^) and remifentanil (1–2 ng mL^–1^), keeping Ramsay score 2–3. Oxygen was delivered continuously via high-flow humidified nasal cannula (50 L min^–1^, FiO_2_ 0.6–0.8).

The primary endpoint of this study was “anesthetic conversion,” defined as any unplanned conversion from non-intubated spontaneous-ventilation anesthesia (NISVA) to tracheal intubation with controlled ventilation, necessitated by patient-related factors—refractory hypoxemia, hemodynamic collapse, or surgical emergency. Only the placement of a cuffed endotracheal tube and initiation of mechanical ventilation qualified as an anesthetic conversion and was counted toward the primary outcome. When conversion was deemed necessary the following sequence was applied: (1) The patient was returned to the supine position (“turn-back” procedure). (2) Rapid-sequence induction was performed and a left-sided double-lumen tube was inserted while the patient was still in the lateral decubitus position; the table was subsequently re-adjusted as required. (3) One-lung ventilation was commenced and surgery continued.

Surgical conversion, in contrast, referred to intra-operative extension of the thoracoscopic incision or conversion to a limited thoracotomy while spontaneous breathing was preserved and the airway remained un-intubated. Such events were not regarded as anesthetic conversions; patients remained in the NISVA cohort for all analyses and the maneuver was recorded merely as an operative adjustment.

### 2.4 Statistical analysis

Data analysis was performed using R software (version 4.2.1) and SPSS 22.0. Categorical variables were reported as frequencies and percentages, while continuous variables were reported as mean ± standard deviation (SD) or median with interquartile range (IQR), depending on the distribution characteristics of the data. Comparisons of categorical variables were conducted using chi-square tests or Fisher’s exact tests, while comparisons of continuous variables were performed using Student’s *t*-tests or Mann-Whitney U tests. Variable selection was completed through LASSO regression (glmnet package) and univariate logistic regression (*P* < 0.2). The predictive model was constructed based on multivariate logistic regression (stepwise AIC backward selection, glm package). A nomogram was generated using the rms package. Model validation was conducted through Bootstrap resampling (1,000 times) and internal validation set. Model performance was evaluated through receiver operating characteristic (ROC) curve analysis [with area under the curve (AUC) as the primary indicator], calibration curves (Hosmer-Lemeshow test), and decision curve analysis (DCA), which were used to assess the model’s discrimination ability, calibration ability, and clinical utility, respectively. The AUC grading criteria were as follows: low (0.5–0.7), moderate (0.7–0.9), and high (0.9–1.0). A two-sided *P* < 0.05 was considered statistically significant.

## 3 Results

### 3.1 Baseline information

A total of 244 patients who underwent pulmonary surgery under NISVA were included in this study. The overall incidence of Intraoperative Endotracheal Intubation (IETI) was 45.49% (111/244). Patients were randomly divided into a training set (*N* = 170) and a validation set (*N* = 74) in a 7:3 ratio. No statistically significant differences were observed between the two groups in terms of gender, age, BMI, chronic obstructive pulmonary disease (COPD), hypertension, cardiovascular disease, personal history of cerebrovascular accident (PHCI), diabetes, thyroid function, allergy history, smoking history, asthma history, difficult airway, Type of surgery (sublobar resection vs. lobectomy), surgical site (upper/middle lobe vs. lower lobe), surgical duration, intraoperative blood loss, preoperative hypoxemia, regional cerebral oxygen saturation (rScO_2_), C-reactive protein, albumin, and white blood cell count (all *P* > 0.05). The balance between groups was satisfactory (Table 1), meeting the requirements for predictive model construction.

**TABLE 1:** Baseline characteristics of the study patients.

Characteristics	Total (*N* = 244)	Training group (*N* = 170)	Validation group (*N* = 74)	*P*- value
IETI, n (%)				0.815
Non-occurrence	133 (54.51%)	94 (55.29%)	39 (52.70%)	
Occurrence	111 (45.49%)	76 (44.71%)	35 (47.30%)	
Difficult airway, n (%)				0.787
No	104 (42.62%)	71 (41.76%)	33 (44.59%)	
Yes	140 (57.38%)	99 (58.24%)	41 (55.41%)	
Gender, n (%)				0.803
Female	120 (49.18%)	85 (50.00%)	35 (47.30%)	
Male	124 (50.82%)	85 (50.00%)	39 (52.70%)	
Age (years), n (%)				0.355
<60	128 (52.46%)	93 (54.71%)	35 (47.30%)	
≥ 60	116 (47.54%)	77 (45.29%)	39 (52.70%)	
COPD				0.346
No	129 (52.87%)	86 (50.59%)	43 (58.11%)	
Yes	115 (47.13%)	84 (49.41%)	31 (41.89%)	
Smoking history, n (%)				0.094
No	159 (65.16%)	117 (68.82%)	42 (56.76%)	
Yes	85 (34.84%)	53 (31.18%)	32 (43.24%)	
BMI (kg/m^2^)	26.03 (21.52;30.49)	26.09 (21.52;30.05)	25.93 (21.84;31.24)	0.778
Hypertension, n (%)				0.696
No	161 (65.98%)	114 (67.06%)	47 (63.51%)	
Yes	83 (34.02%)	56 (32.94%)	27 (36.49%)	
Type of surgery, n (%)				0.943
Sublobectomy	136 (55.74%)	94 (55.29%)	42 (56.76%)	
Lobectomy	108 (44.26%)	76 (44.71%)	32 (43.24%)	
Cardiovascular disease, n (%)				0.842
No	118 (48.36%)	81 (47.65%)	37 (50.00%)	
Yes	126 (51.64%)	89 (52.35%)	37 (50.00%)	
PHCI, n (%)				0.244
No	133 (54.51%)	88 (51.76%)	45 (60.81%)	
Yes	111 (45.49%)	82 (48.24%)	29 (39.19%)	
Surgical site, n (%)				0.921
Upper/middle lobe	138 (56.56%)	97 (57.06%)	41 (55.41%)	
Lower lobe	106 (43.44%)	73 (42.94%)	33 (44.59%)	
Thyroid function, n (%)				0.825
Normal	146 (59.84%)	103 (60.59%)	43 (58.11%)	
Abnormal	98 (40.16%)	67 (39.41%)	31 (41.89%)	
Allergy history, n (%)				0.764
No	130 (53.28%)	89 (52.35%)	41 (55.41%)	
Yes	114 (46.72%)	81 (47.65%)	33 (44.59%)	
Diabetes mellitus, n (%)				0.984
No	130 (53.28%)	90 (52.94%)	40 (54.05%)	
Yes	114 (46.72%)	80 (47.06%)	34 (45.95%)	
Type of lung cancer, n (%)				0.854
Squamous cell carcinoma	139 (56.97%)	98 (57.65%)	41 (55.41%)	
Adenocarcinoma	105 (43.03%)	72 (42.35%)	33 (44.59%)	
Duration of surgery, n (%)				0.171
<3	175 (71.72%)	117 (68.82%)	58 (78.38%)	
≥ 3	69 (28.28%)	53 (31.18%)	16 (21.62%)	
rScO_2_, (%)	73.00 (66.00;79.00)	72.50 (67.00;79.00)	73.00 (65.25;80.75)	0.807
Intraoperative blood loss, (mL)	73.00 (65.00;80.00)	72.00 (65.00;80.00)	73.50 (67.00;79.00)	0.337
Preoperative hypoxemia, n (%)				0.116
No	142 (58.20%)	105 (61.76%)	37 (50.00%)	
Yes	102 (41.80%)	65 (38.24%)	37 (50.00%)	
History of asthma, n (%)				1.000
No	129 (52.87%)	90 (52.94%)	39 (52.70%)	
Yes	115 (47.13%)	80 (47.06%)	35 (47.30%)	
C-reactive protein, (mg/L)	21.54 (11.48;30.75)	22.04 (11.65;30.37)	20.22 (10.72;31.67)	0.814
Preoperative albumin, (g/L)	37.00 (31.00;41.00)	37.00 (31.00;41.00)	37.00 (32.00;40.75)	0.712
WBC, (× 10^9^/L)	10.00 (7.00;12.25)	10.00 (7.00;13.00)	10.00 (8.00;12.00)	0.602

### 3.2 Variable selection

**FIGURE 2:**
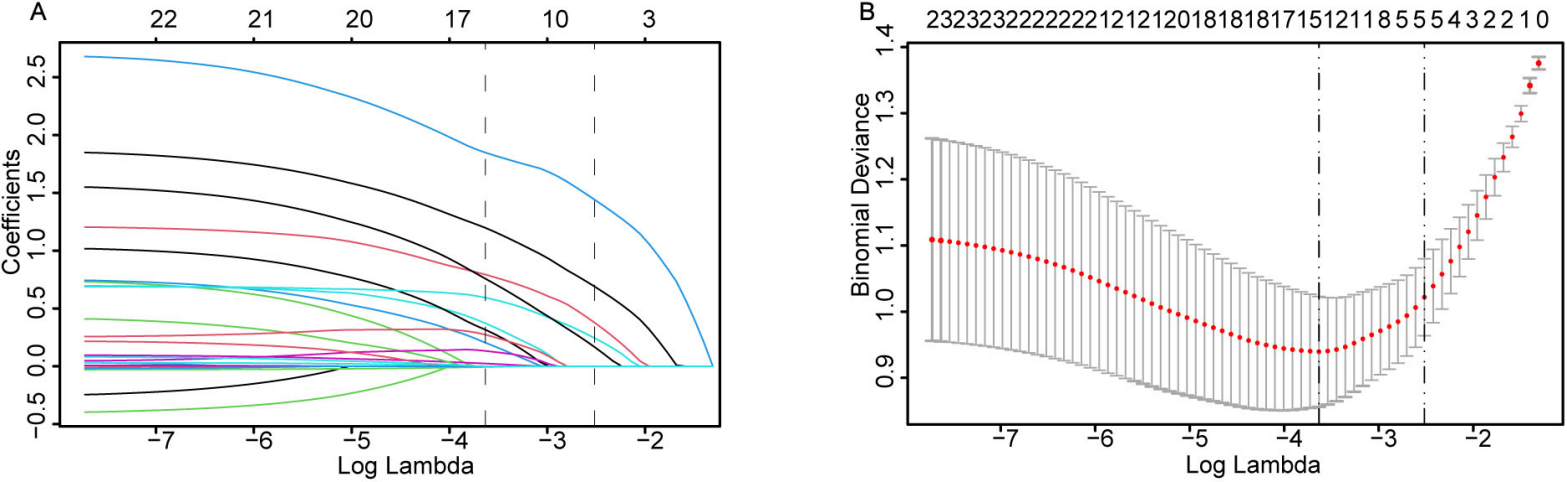
LASSO regression for variable selection and parameter tuning. **(A)** As the log-penalty strength (log λ) increases, the number of retained variables shrinks. **(B)** Ten-fold cross-validation identifies the optimal λ at the 1-SE rule (dashed vertical line), yielding a parsimonious yet predictive model.

LASSO regression with 10-fold cross-validation (using the lambda.1se criterion) was performed for dimensionality reduction and feature selection from the initial set of 23 predictor variables, with the variable shrinkage path demonstrated in Figure 2A and the cross-validation curve presented in Figure 2B, to optimize model parsimony while preserving predictive performance. The key predictors retained after screening were identified. Univariate and multivariate logistic regression analyses (Table 2) revealed that preoperative hypoxemia (OR = 2.973, 95% CI 1.249–7.340, *P* = 0.015), surgical site (OR = 2.462, 95% CI 1.055– 5.827, *P* = 0.037), Type of surgery (OR = 3.600, 95% CI 1.575–8.559, *P* = 0.003), difficult airway (OR = 4.708, 95% CI 1.984–11.87, *P* = 0.001), and surgical duration ≥ 3 h (OR = 11.81, 95% CI 4.617–33.96, *P* < 0.001) were independent risk factors for conversion to endotracheal intubation during pulmonary surgery under NISVA. These factors were statistically significant in the multivariate analysis, indicating a significant association with the event of conversion to intubation. The final multivariate logistic regression model included five independent predictors. The regression coefficients (β), standard errors, and intercept of the final model are presented in Table 3 to facilitate model reproducibility and external validation.

**TABLE 2:** Univariate and multivariate analyses of risk factors for IETI during NISVA lung surgery.

Characteristics	Univariate analysis		Multivariate analysis	
	OR (95%CI)	*P*-value	OR (95%CI)	*P*-value
WBC	1.03 (0.946–1.124)	0.496		
Preoperative albumin	0.991 (0.935–1.051)	0.765		
C-reactive protein	0.985 (0.958–1.013)	0.291		
History of asthma	1.363 (0.743–2.509)	0.318		
Preoperative hypoxemia	3.067 (1.626–5.891)	0.001	2.973 (1.249–7.340)	0.015
Intraoperative blood loss	1.014 (0.978–1.052)	0.456		
rScO_2_	1.011 (0.971–1.052)	0.599		
Duration of surgery	15.60 (6.939–39.15)	< 0.001	11.81 (4.617–33.96)	< 0.001
Type of lung cancer	1.193 (0.647–2.203)	0.572		
Diabetes mellitus	1.82 (0.991–3.375)	0.055		
Allergy history	1.078 (0.588–1.978)	0.808		
Thyroid function	2.757 (1.472–5.249)	0.002		
Surgical site	4.239 (2.243–8.203)	< 0.001	2.462 (1.055–5.827)	0.037
PHCI	1.668 (0.909–3.084)	0.100		
Cardiovascular disease	1.36 (0.742–2.507)	0.322		
Type of surgery	4.041(2.147–7.78)	< 0.001	3.600 (1.575–8.559)	0.003
Hypertension	1.531 (0.805–2.926)	0.194		
BMI	1.083 (1.017–1.155)	0.014		
Smoking history	1.8 (0.937–3.488)	0.079		
COPD	2.047 (1.112–3.811)	0.022		
Age	0.872 (0.473–1.601)	0.659		
Gender	0.51 (0.274–0.939)	0.032		
Difficult airway	6.822 (3.422–14.32)	< 0.001	4.708 (1.984–11.87)	0.001

**TABLE 3:** Complete regression coefficients of the final multivariate logistic model for predicting intraoperative conversion to endotracheal intubation.

Variable	Category/definition	β Coefficient	Standard Error	Odds Ratio (95% CI)	*P*-value
Intercept	–	−3.85	0.64	–	< 0.001
Preoperative hypoxemia	SpO_2_ ≤ 92% or PaO_2_ < 60 mmHg	1.09	0.45	2.97 (1.25–7.34)	0.015
Surgical site	Lower lobe (vs. upper/middle lobe)	0.9	0.43	2.46 (1.06–5.83)	0.037
Type of surgery	Lobectomy (vs. sublobectomy)	1.28	0.43	3.60 (1.58–8.56)	0.003
Difficult airway	DARS ≥ 1	1.55	0.46	4.71 (1.98–11.87)	0.001
Surgical duration	≥ 3 h (vs. < 3 h)	2.47	0.52	11.81 (4.62–33.96)	< 0.001

The model equation is: Log-odds (IETI) = −3.85 + (1.09 × Preoperative hypoxemia) + (0.90 × Surgical site) + (1.28 × Type of surgery) + (1.55 × Difficult airway) + (2.47 × Surgical duration).

### 3.3 *Post hoc* analysis of pulmonary function

Given that forced expiratory volume in the first second (FEV1) data were missing in 93 of 244 patients (38.1%), we conducted a sensitivity analysis to evaluate its potential association with intraoperative endotracheal intubation (IETI). Among the 151 patients (61.9%) with available preoperative spirometry, FEV1% predicted and FEV1 < 70% predicted were tested in univariate and multivariate logistic regression models. Neither FEV1% predicted (OR = 0.994, 95% CI: 0.978–1.011, *P* = 0.18) nor FEV1 < 70% predicted (OR = 1.21, 95% CI: 0.66–2.23, *P* = 0.54) were significantly associated with IETI. Furthermore, incorporating FEV1 into the final multivariate model did not improve discriminative performance (AUC increased by < 0.01). Therefore, FEV1 was not included in the final nomogram. Detailed FEV1 data are presented in Supplementary Table 1.

### 3.4 Model construction and validation

**FIGURE 3:**
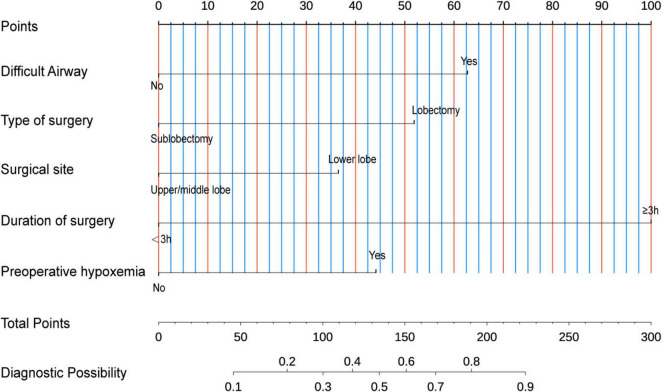
Nomogram for predicting intraoperative conversion to intubation in patients undergoing lung surgery under Non-intubated spontaneous ventilation anesthesia (NISVA). IETI, intraoperative endotracheal intubation; Difficult Airway, predicted difficult airway; Lobectomy, lobectomy vs. non-lobectomy; Lower lobe, lower-lobe vs. upper/middle-lobe surgery; Duration of surgery, operative time; Preoperative hypoxemia, baseline SpO_2_ < 90%. (Example: A representative case with preoperative hypoxemia (20 points), lower lobe lobectomy (40 points total), difficult airway (30 points), and duration ≥ 3 h (40 points) achieved a total score of 130 points on the nomogram, corresponding to a predicted IETI probability of 70%.).

In the model construction phase, we developed a nomogram prediction model based on the identified independent risk factors (Figure 3). The model incorporated six key variables: preoperative hypoxemia, abnormal thyroid function, surgical site, Type of surgery, difficult airway, and surgical duration ≥ 3 h. Each variable was assigned a specific weight according to its contribution to the risk of conversion to intubation. The total score was obtained by summing the weights, which was then mapped to the axis of diagnostic probability to achieve individualized prediction of the risk of conversion to endotracheal intubation during pulmonary surgery under NISVA.

**FIGURE 4:**
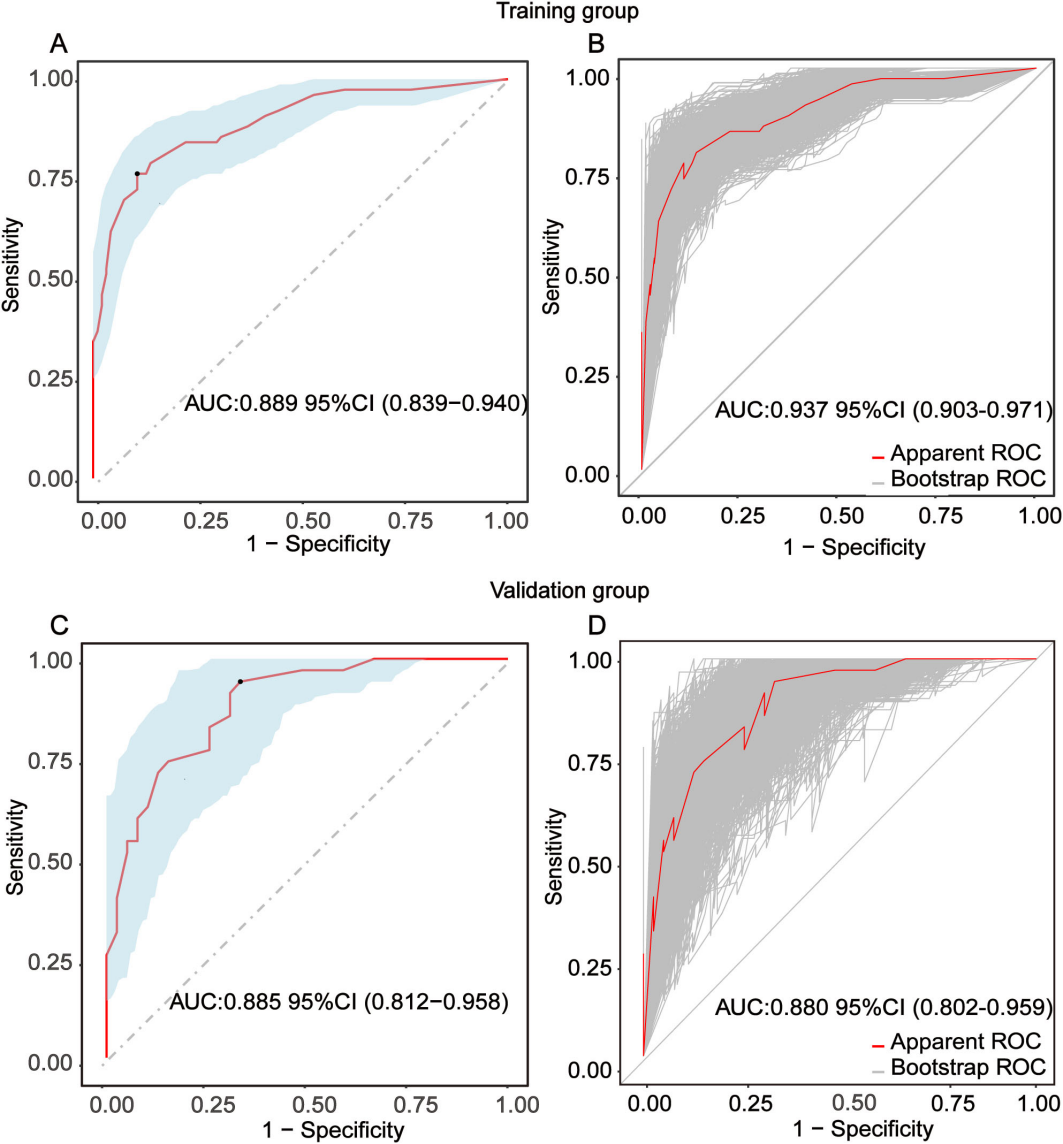
Receiver-operating-characteristic (ROC) evaluation of the nomogram for predicting intra-operative conversion to endotracheal intubation (IETI). **(A)** Apparent ROC curve derived from the training group. **(B)** Internally validated ROC curve obtained by 1,000-bootstrap resampling in the training group. **(C)** Apparent ROC curve generated from the independent validation group. **(D)** Internally validated ROC curve based on 1000-bootstrap resampling in the validation group.

**FIGURE 5:**
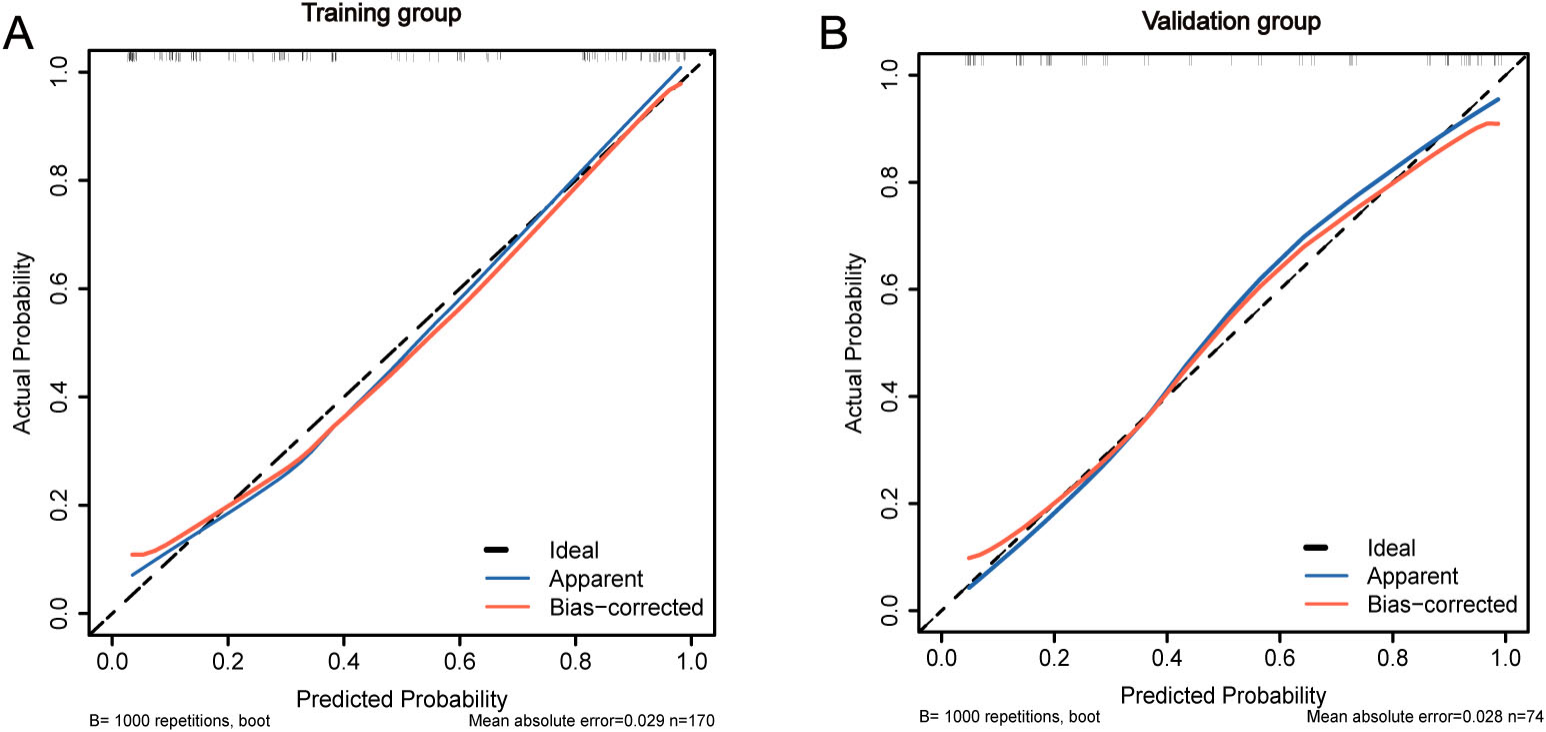
Calibration performance of the IETI-prediction nomogram. **(A)** Validation via bootstrap resampling (1,000 iterations) and a calibration curve of the training group nomogram model. **(B)** Validation via bootstrap resampling (1,000 iterations) and calibration curve of the validation group nomogram model.

**FIGURE 6:**
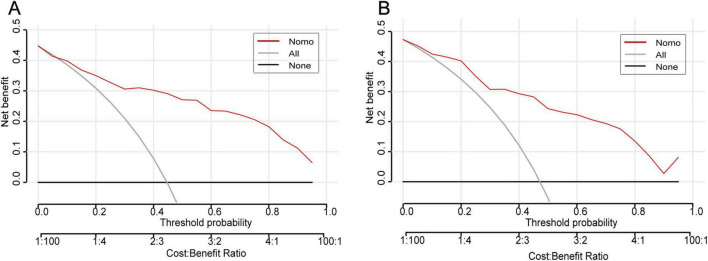
Decision-curve analysis of the IETI-prediction nomogram. **(A)** Training cohort; **(B)** validation cohort. Within threshold probabilities of approximately 5–85%, applying the nomogram to guide the decision for intra-operative conversion to endotracheal intubation yields a higher net clinical benefit than either the “intubate-all” or the “intubate-none” strategy.

**FIGURE 7:**
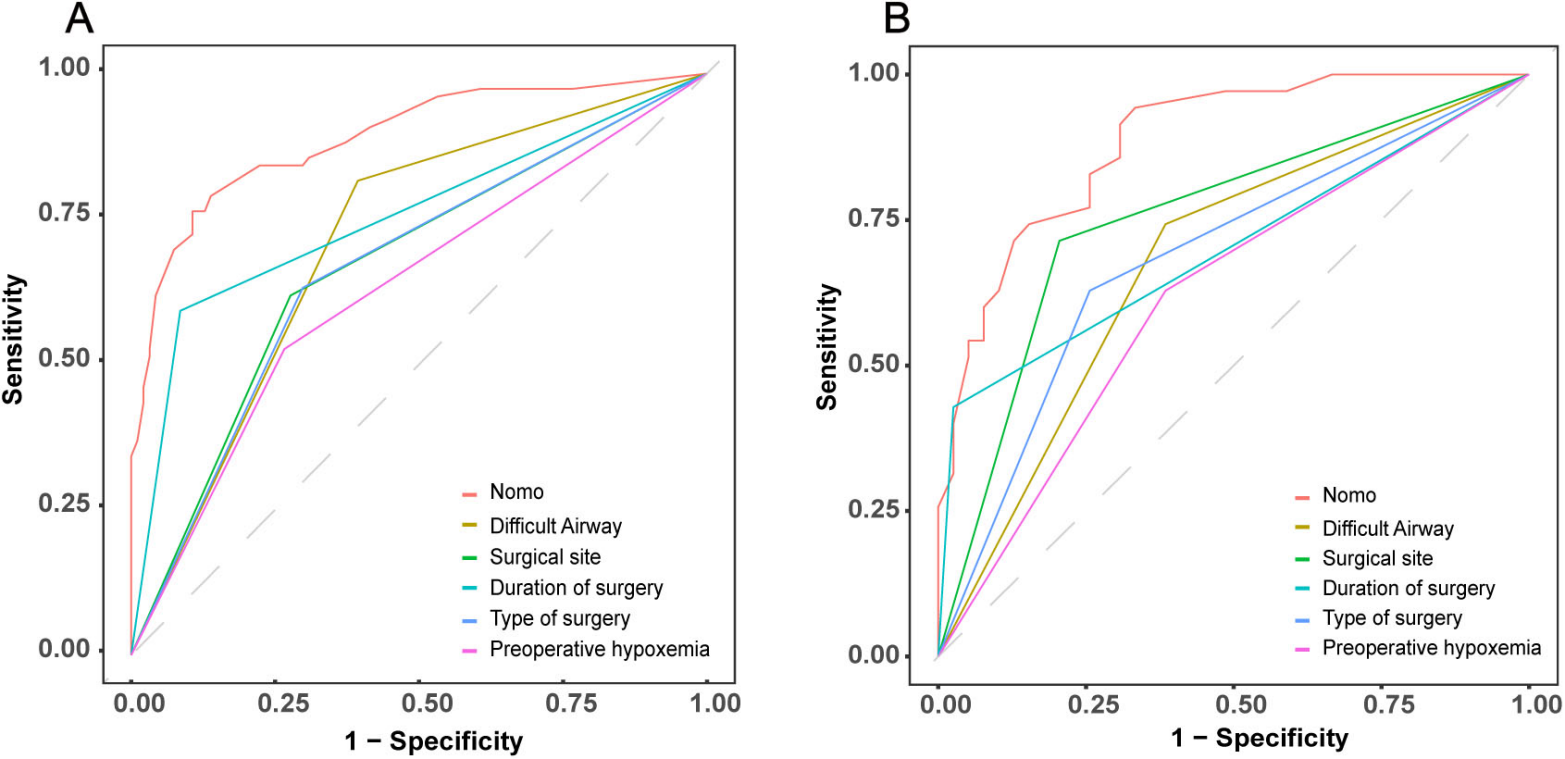
Comparative discriminative performance of the IETI-prediction nomogram versus individual clinical indicators. ROC curves for the nomogram and each standalone risk factor are plotted for the training cohort **(A)** and the validation cohort **(B)**.

To evaluate the discriminative performance of the nomogram, we constructed receiver operating characteristic (ROC) curves for both cohorts. The apparent AUCs were 0.889 (95% CI 0.839–0.940) and 0.885 (95% CI 0.812–0.958) for the training and validation sets, respectively (Figures 4A, C). After 1,000 bootstrap resamples, the internally validated AUCs remained high at 0.937 (95% CI 0.903–0.971) and 0.880 (95% CI 0.802–0.959) (Figures 4B, D), indicating robust and stable discrimination capacity of the model. Second, calibration curves for the training and validation sets were generated using bootstrap sampling (Figures 5A, B), and the results showed good agreement between predicted and actual probabilities, with mean absolute errors of 0.029 (*n* = 170) for the training set and 0.028 (*n* = 74) for the validation set. Third, decision curve analysis (DCA) demonstrated that the nomogram model had higher net benefit than the “all” and “none” strategies within a certain threshold range (Figures 6A, B), indicating its clinical utility within these thresholds. Finally, subgroup analysis showed that the ROC curves of the model for variables such as difficult airway, surgical site, surgical duration, Type of surgery, and preoperative hypoxemia were higher than the sensitivity and specificity of different subgroup variables in both the training and validation sets (Figures 7A, B), further validating the model’s predictive performance across different subgroups.

## 4 Discussion

This study focuses on the development and evaluation of a predictive model for the risk of Intraoperative Endotracheal Intubation (IETI) during pulmonary resection under NISVA. Through rigorous statistical methods and data analysis, we successfully developed a nomogram model based on multivariate logistic regression, identifying five key independent risk factors: preoperative hypoxemia, surgical site, Type of surgery, difficult airway, and surgical duration ≥ 3 h. The model’s performance was validated in terms of discrimination, calibration, and clinical utility.

Preoperative hypoxemia is a significant precipitating factor for IETI. Previous studies have indicated that patients with this condition have limited pulmonary function reserves and struggle to maintain adequate oxygenation under NISVA (9, 10). Our study further corroborates this finding, demonstrating that patients with preoperative hypoxemia are more prone to worsening intraoperative hypoxemia under NISVA, thereby significantly increasing the risk of conversion to endotracheal intubation. Similarly, differences in surgical site are crucial. Previous research has highlighted that lower lobe surgeries, due to their anatomical complexity and limited operative space (11, 12), are more likely to induce hypoxemia and intraoperative complications (13). Consistent with these findings, our study revealed a significantly higher risk of IETI in lower lobe surgeries compared to upper or middle lobe surgeries, which is closely related to the anatomical characteristics and surgical complexity.

It should be emphasized that the high conversion rate observed in our cohort does not imply that major pulmonary resection is inherently incompatible with spontaneous ventilation. However, regarding type of surgery, previous studies have shown that lobectomy, compared to sublobar resection, involves a larger surgical scope and more complex procedures, imposing a greater physiological impact on patients and leading to intraoperative fluctuations in physiological parameters (14–16). Our study similarly found that lobectomy significantly increased the incidence of IETI, consistent with the substantial impact of surgical scope and complexity on patients’ respiratory and circulatory systems. Management of difficult airways has always been a clinical challenge, and previous studies have also indicated that difficult airways significantly increase the risk of conversion to endotracheal intubation (17–19). Our study reaffirms this, showing that difficult airways are hard to manage effectively under NISVA, leading to inadequate ventilation and poor oxygenation, necessitating conversion to endotracheal intubation for surgical safety.

The influence of surgical duration is also noteworthy. Previous studies have shown that prolonged pulmonary surgeries significantly increase the physiological burden on patients, leading to gradual deterioration of oxygenation and ventilation functions (20, 21). Our study found that a surgical duration of ≥ 3 h is an independent risk factor for IETI, closely related to the depletion of patients’ physiological reserves and the deterioration of physiological parameters during long surgeries.

In terms of model validation, receiver operating characteristic (ROC) curve analysis showed that the AUC for the training set and validation set reached 0.889 and 0.880, respectively, indicating that the model has strong discrimination ability to accurately distinguish between high- and low-risk patient groups. Calibration curves further confirmed a high degree of consistency between predicted and actual probabilities, with mean absolute errors of only 0.029 for the training set and 0.028 for the validation set, highlighting the reliability of the model’s predictions. Decision curve analysis revealed that within the critical threshold range for clinical decision-making, the model’s net benefit significantly surpassed that of the “all” or “none” strategies, fully demonstrating its value in clinical practice. It can assist doctors in formulating personalized response plans in advance and optimizing preoperative assessment and anesthesia planning.

In this cohort the incidence of intraoperative endotracheal intubation reached 45.49% (111/244), a figure that surpasses most contemporary reports; nevertheless, this elevation is neither incidental nor uncontrolled. To begin with, the study population was deliberately enriched for high-risk phenotypes: not only did 41.8% of participants arrive with pre-operative hypoxemia (SpO_2_ ≤ 92%), an independent predictor of conversion (OR 2.97, *P* = 0.015), but moreover 57.4% exhibited a difficult airway (DARS ≥ 1, OR 4.71, *P* = 0.001), while furthermore 44.3% required anatomical lobectomy with its attendant parenchymal manipulation and prolonged lung retraction. Consequently, the cumulative burden of these anatomical and physiological vulnerabilities constitutes the primary driver of the observed rate. In addition, the protocol imposed an intentionally low threshold for conversion: intubation was triggered whenever SpO_2_ remained < 90% for more than 5 min despite FiO_2_ 1.0, or whenever MAP persisted below 60 mmHg for at least 3 min after exclusion of anesthetic excess or hypovolemic. Thus, although this conservative algorithm inevitably increased the conversion frequency, it simultaneously precluded catastrophic hypoxemia or circulatory collapse, thereby upholding the principle of safety primacy throughout the procedure.

The incidence of airway trauma related to double-lumen endobronchial tube (DLT) insertion ranges from 0.2 to 2 ‰; severe complications—vocal-cord hematoma, cricoarytenoid dislocation and membranous tracheal tear—are significantly associated with repeated intubation attempts, oversized catheter selection and the narrower female airway (cricoid diameter 12.0–12.2 mm) (22). Non-intubated spontaneous ventilation anesthesia (NISVA) eliminates this hazard by avoiding intubation, but must be balanced against a restricted operative field due to partial ventilation of the operated lung. Should emergency conversion become necessary, the higher driving pressure required for rescue one-lung ventilation may offset the initial protective effect (23). Future randomized trials should therefore compare NISVA with conventional OLV in terms of mechanical power, inflammatory markers (IL-6, TNF-α) and postoperative CT atelectasis scores to quantify its lung-protective benefit. Although FEV1 is a standard index of preoperative pulmonary function, it was unavailable in 38.1% of our cohort (93/244), mainly because of local insurance restrictions and patients’ inability to cooperate with spirometry. In the 151 individuals (61.9%) with usable data we performed a *post hoc* sensitivity analysis. Neither FEV1% predicted (OR = 0.994, 95% CI: 0.978–1.011, *P* = 0.18) nor FEV1 < 70% predicted (OR = 1.21, 95% CI: 0.66–2.23, *P* = 0.54) was significantly associated with intraoperative endotracheal intubation (IETI). Adding FEV1 to the multivariate model increased the AUC by < 0.01, indicating no meaningful gain in discrimination. We hypothesize that the lack of association may reflect: (1) that conversion was driven by acute physiological derangement (e.g., hypoxemia, hemodynamic instability) rather than baseline pulmonary function; and (2) that NISVA tolerates impaired ventilatory capacity, allowing even patients with moderate-to-severe obstruction to complete surgery safely. Thus, although FEV1 remains clinically important, its independent predictive value for IETI was limited in the present context and it was not retained in the final nomogram. This finding suggests the model’s potential clinical utility may be most relevant in comparable high-risk populations. Furthermore, several unmeasured confounders—including variations in anesthesiologist expertise, specific airway management devices, ventilation strategies, and severity of comorbid conditions such as chronic obstructive pulmonary disease (COPD)—may have influenced the outcomes and warrant further investigation.

However, the limitations of this study must not be overlooked. The single-center, retrospective design is prone to selection and confounding biases, and the limited sample size also restricts in-depth analysis of rare factors and complex interactions. Future research urgently needs to conduct multicenter, large-sample prospective studies to continuously improve and optimize the model structure. Meanwhile, the current model has relatively limited variable inclusion, and there are still potential influencing factors that need to be further explored and integrated to comprehensively enhance the model’s predictive accuracy.

Overall, the predictive model developed in this study provides clinicians with a powerful tool for individualized risk assessment, which is expected to significantly improve the quality of perioperative management and optimize patient outcomes. Future multicenter prospective trials will integrate pre-operative pulmonary function (FEV1, DLCO), 6-min-walk distance, and quantitative CT lung volumes to further refine risk stratification and externally validate the model. The prediction model developed in this study can be integrated into preoperative workflows to enable individualized risk assessment. By inputting key preoperative variables into the nomogram, clinicians can quantify IETI risk and optimize anesthesia management, thereby improving perioperative outcomes. Future work will focus on model validation and mobile application development to enhance clinical translation.

## Data Availability

The original contributions presented in this study are included in this article/Supplementary material, further inquiries can be directed to the corresponding author.
