# Supplementary material for: A nomogram for predicting intra-operative conversion to endotracheal intubation during non-intubated spontaneous ventilation anesthesia in pulmonary resection: development of a risk prediction model in hypoxic and high-risk patients
Source: Front Med (Lausanne). 2025 Nov 28;12:1709129. doi: 10.3389/fmed.2025.1709129 (PMC12698436; doi:10.3389/fmed.2025.1709129)
Supplement: Supplementary file 1 [file Table_1.docx]

**Supplementary Table 1**
Preoperative FEV₁ data in patients with available spirometry (n = 151).

| **Variable** | **Total (n = 151)** | **Training group (n = 105)** | **Validation group (n = 46)** | **P value** |
| --- | --- | --- | --- | --- |
| FEV₁ median, (L) | 1.89 [1.52–2.31] | 1.91 [1.55–2.33] | 1.85 [1.48–2.29] | 0.572 |
| FEV₁ % predicted, median [IQR] | 78.3 [65.4–89.7] | 79.1 [66.2–90.5] | 77.5 [64.8–88.9] | 0.618 |
| FEV₁ < 70% predicted, n (%) | 48 (31.8) | 32 (30.5) | 16 (34.8) | 0.609 |

Note: FEV₁ measurements were obtainable in 151 of 244 patients (61.9%). There were no significant differences between the training and validation subsets.
